# Regulation of floral stem cell termination in *Arabidopsis*

**DOI:** 10.3389/fpls.2015.00017

**Published:** 2015-02-02

**Authors:** Bo Sun, Toshiro Ito

**Affiliations:** ^1^Temasek Life Sciences Laboratory, 1 Research Link, National University of SingaporeSingapore; ^2^Department of Biological Sciences, National University of SingaporeSingapore

**Keywords:** *Arabidopsis*, floral meristem, stem cell, determinacy, flower development

## Abstract

In *Arabidopsis*, floral stem cells are maintained only at the initial stages of flower development, and they are terminated at a specific time to ensure proper development of the reproductive organs. Floral stem cell termination is a dynamic and multi-step process involving many transcription factors, chromatin remodeling factors and signaling pathways. In this review, we discuss the mechanisms involved in floral stem cell maintenance and termination, highlighting the interplay between transcriptional regulation and epigenetic machinery in the control of specific floral developmental genes. In addition, we discuss additional factors involved in floral stem cell regulation, with the goal of untangling the complexity of the floral stem cell regulatory network.

## Introduction

The flower is an elegant structure produced by angiosperms for effective reproduction. In *Arabidopsis*, floral organs are built in four whorls of concentric circles. From outermost to innermost, they consist of four sepals, four petals, six stamens and two fused carpels. The molecular mechanism specifying the identity of each whorl of floral organs is explained by the genetic ABCE model (Krizek and Fletcher, 2005). All four whorls of floral organs are derived from a self-sustaining stem cell pool named the floral meristem (FM), which arises from the peripheral regions of the shoot apical meristem (SAM). Much like the stem cells in the SAM, the stem cells in the FM are maintained by a signaling pathway involving the homeodomain protein WUSCHEL (WUS) and the CLAVATA (CLV) ligand-receptor system (Fletcher et al., 1999; Brand et al., 2000; Schoof et al., 2000). *WUS* is expressed in the organizing center, and it specifies and maintains the stem cell identity of the overlying cells. Expansion of *WUS* expression is prevented by the CLV signaling pathway, in which the CLV3 peptide is transcriptionally induced by WUS in the stem cells (Yadav et al., 2011; Daum et al., 2014). Due to the negative feedback regulatory loop of *CLV3* and *WUS*, the stem cell pool remains constant in the initial floral developmental stages (stage 1~2) (Smyth et al., 1990; Schoof et al., 2000).

**Figure 1:**
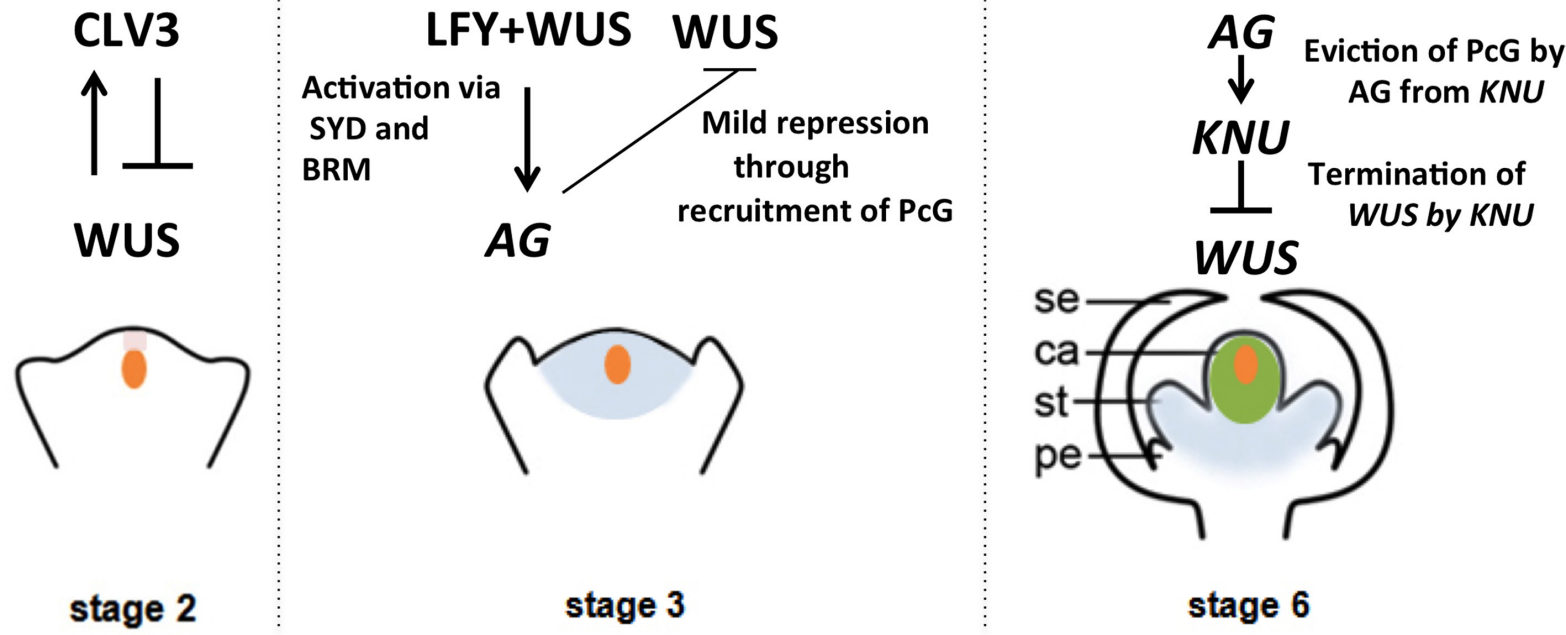
**Regulation of the timing of floral stem cell termination**. Signaling cascades, transcriptional regulation and epigenetic regulation of the key proteins involved in floral meristem regulation are illustrated in a stage-specific manner. Orange indicates the domain of *WUS* expression, and pink, blue and green indicate the expression domains of *CLV3, AG*, and *KNU*, respectively.

In the stage 3 floral bud, the C class gene *AGAMOUS (AG)* is induced by LEAFY (LFY) together with WUS in whorls 3 and 4 (Lenhard et al., 2001; Lohmann et al., 2001). AG has two major roles. It specifies reproductive organs, and it also regulates floral stem cell activity (Lenhard et al., 2001; Lohmann et al., 2001). In stage 6, floral stem cells are terminated in an AG-dependent manner to ensure proper development of the carpels. With respect to floral stem cell regulation, the major two pathways, the *AG-WUS* pathway and the *CLV-WUS* pathway, seem to function independently. The double mutant *ag clv1* shows an additive phenotype of *ag* and *clv1*, and it expresses *WUS* in a broader domain than the *ag* mutant flower (Lohmann et al., 2001). In fact, the *CLV-WUS* pathway regulates floral stem cells spatially to restrict and maintain the stem cell pool in the early floral stages (stage 1–6), whereas the *AG-WUS* pathway provides temporal regulation to shut off stem cell activity at floral stage 6 (Figure 1). The precise timing of *WUS* repression is a key factor that determines the number of cells produced for reproductive organ development.

## Direct and indirect roles of AG in *WUS* repression

AG is reported to directly bind to the *WUS* locus to repress *WUS* expression (Liu et al., 2011). Based on an ethyl methanesulfonate mutagenesis screening of enhancer mutants of a weak allele, *ag-10*, which has only a moderate effect on floral meristem determinacy, one *CURLY LEAF* (*CLF*) mutant allele, *clf-47*, was identified (Liu et al., 2011). This suggests that *CLF* is required for floral meristem determinacy. CLF is a core component of polycomb repressive complex 2 (PRC2), which suggests that *WUS* repression is associated with the deposition of the repressive mark H3 lysine 27 tri-methylation (H3K27me3), a mark that is mediated by the polycomb group proteins (PcG). Consistent with this, one mutant allele of *TERMINAL FLOWER 2* (*TFL2*), a PRC1 factor in *Arabidopsis*, can enhance the *ag-10* indeterminate phenotype (Liu et al., 2011). The *ag-10 tfl2-2* double mutant flowers show enlarged carpels bearing ectopic internal organs, as observed in *ag-10 clf-47*. These results indicate that *WUS* is a target of PcG during flower development. AG binds to the two CArG boxes in the *WUS* 3′ non-coding region, and TFL2 occupancy at *WUS* is largely compromised in the *ag-1* null mutant background. These results suggest that AG has a role in the recruitment of PcG to repress *WUS*. However, whether AG recruits PcG directly is still an open question.

*35S::AG* transgenic plants do not show any obvious floral meristem defects (Mizukami and Ma, 1997), and *WUS* is only mildly repressed after stage 3 directly by AG. For the termination of *WUS* at floral stage 6, a C2H2 zinc finger repressor protein, KNUCKLES (KNU), plays a pivotal role (Payne et al., 2004; Sun et al., 2009). *KNU* expression starts in stage 5–6, and mutation of *KNU* leads to enlarged carpels and repeated ectopic growth of stamens and carpels. This indeterminate floral phenotype is caused by the prolonged activity of *WUS*, showing that KNU is necessary for floral stem cell termination. *KNU* is directly induced by AG, and mutations in three CArG box sequences on the *KNU* promoter can abolish *KNU* induction (Sun et al., 2009). Timed induction of *KNU* by AG in stage 6 of flower development ensures floral meristem termination and proper development of the female reproductive organs. The timing of *KNU* expression is important for balancing floral stem cell proliferation and differentiation. Delayed *KNU* expression leads to indeterminate flowers with more stamens, and ectopic *KNU* activity can terminate floral meristem precociously and produce flowers without carpels. *KNU* is also regulated by PcG-mediated H3K27me3, and the removal of the repressive marks of H3K27me3 is AG-dependent. It takes approximately 2 days for AG to induce *KNU* in stage 6. During these 2 days, the H3K27me3 level on the *KNU* locus is progressively reduced, revealing a potential link between the transcriptional activation of *KNU* by AG and AG-dependent removal of H3K27me3 from the *KNU* chromatin (Sun et al., 2009).

## Epigenetic regulation of termination timing in floral stem cells

In floral meristems, cell division take 1–2 days on average (Reddy et al., 2004). Therefore, the 2-day of delay in *KNU* induction corresponds to 1–2 rounds of cell division. Through cell division, the pre-existing H3K27me3 on the *KNU* locus may be passively diluted by incorporation of unmodified histone H3, enabling *KNU* expression (Sun et al., 2014). The core components of PcG, FIE and EMF2 are associated with specific promoter regions of *KNU*, which include the binding sites of AG. Indeed, this region contains a 153 bp fragment that is the minimal sequence of a functional polycomb response element (PRE). This sequence is both necessary and sufficient for PcG-mediated silencing of a ubiquitous promoter. This raises the possibility that AG plays a role in removing PcG to activate *KNU*. By simulating AG's physical blocking of the site with an artificially-designed TAL protein (a effector-based synthetic DNA binding protein designed to recognize the sequences around the first AG binding site), we showed that a YFP reporter could be activated in a cell cycle-dependent manner, even though it had been silenced by the minimal PRE sequence.

PRE was first identified in the fruit fly *Drosophila*, and it is targeted by the Pho-repressive complex (PhoRC) (Muller and Kassis, 2006). In *Arabidopsis*, homologs of PhoRC have not been identified, but in a genome-wide analysis of FIE binding sites, GA-repeat motifs appeared frequently, much like the *Drosophila* PRE (Deng et al., 2013). The *KNU* PRE is located near the 1kb upstream promoter region of the *KNU* transcriptional start site (Sun et al., 2014). Although the entire *KNU* locus is found to be bound by FIE and EMF2, only the transcribed region is covered by the repressive mark H3K27me3, and the PRE is not covered by the repressive mark. The indispensable role of the *KNU* PRE in recruiting PRC2 and establishing the FIE and EMF2 binding pattern on *KNU* indicates that PcG is first recruited to the *KNU* PRE and may later act on the *KNU* transcribed region to establish the H3K27me3 marks by sliding or by DNA looping. When the AG protein binds to the CArG box sequences that overlap the *KNU* PRE, the occupancy of AG triggers the displacement of PRC2, which leads to the loss of the H3K27me3 marks on *KNU*. Through cell division, H3K27me3 is diluted due to the lack of PcG activity, and *KNU* become de-repressed. Delayed reporter induction has been reported following artificial removal of a PRE by a cre-lox system in *Drosophila*, supporting this model of *KNU* de-repression (Beuchle et al., 2001; Muller et al., 2002).

Alternatively, the H3K27me3 mark can be erased by the JmjC-domain-containing histone demethylases REF6, EFL6, JMJ30 and JMJ32 (Lu et al., 2011; Crevillen et al., 2014; Gan et al., 2014). It has been reported that AG, REF6 and some other MADS-domain proteins may form a large protein complex whose function has not been characterized (Smaczniak et al., 2012). Therefore, it is also possible that, in parallel with H3K27me3 passive dilution, AG may recruit REF6 to the *KNU* promoter to actively remove H3K27me3. However, this hypothesis does not explain why cell cycle progression is required for AG to induce *KNU*. Also, the known mutants for these demethylases show no meristematic defects. Hence, we propose that REF6 might be involved in the regulation of some other direct downstream targets that are induced by AG.

To remove H3K27me3 marks and activate gene expression, other transcription factors or chromatin remodeling factors may perform functions similar to those that AG does. One such example is LFY in the control of the *AG* locus. For *AG* expression, the repressive mark H3K27me3 is removed by LEAFY (LFY), which recruits the SWI/SNF chromatin remodeling factors SPLAYED (SYD) and BRAHMA (BRM) on the *AG* second intron (Wu et al., 2012). Notably, GA-repeat motifs located near PREs are enriched at LFY targets (Wu et al., 2012; Zhang, 2014).

*WUS*, which is required in the organizing center to stimulate the maintenance of stem cell properties in the overlying cells (Yadav et al., 2011; Daum et al., 2014), is negatively regulated by PcG-mediated H3K27me3 (Zhang et al., 2007). In the SAM, the *WUS*-*CLV* signaling pathway works to maintain an appropriately sized stem cell. The signaling pathway remains active in floral stem cells and works to maintain their identity. It is interesting that *WUS* is re-activated and the signaling pathway is re-established in the stage 1 floral primordia (Mayer et al., 1998) and that the SWI/SNF chromatin remodeling factor SYD plays an important role in *WUS* activation (Wagner and Meyerowitz, 2002; Kwon et al., 2005). In floral stage 6, *WUS* is terminated by KNU and later silenced by PcG-mediated H3K27me3 marks (Sun et al., 2009; Liu et al., 2011). Because transcriptional repression of *WUS* and epigenetic silencing of *WUS* both occur at floral stage 6, we suggest that the transcriptional repressor KNU may integrate the two processes. During reproductive development, *WUS* is activated in developing stamens at stages 7-8, and later it is activated in developing ovules (Gross-Hardt et al., 2002; Deyhle et al., 2007). How the repressive mark H3K27me3 is removed from the *WUS* locus in those specific tissues and cell types is another open question that will require further investigation.

## Other factors involved in floral meristem regulation

**Figure 2:**
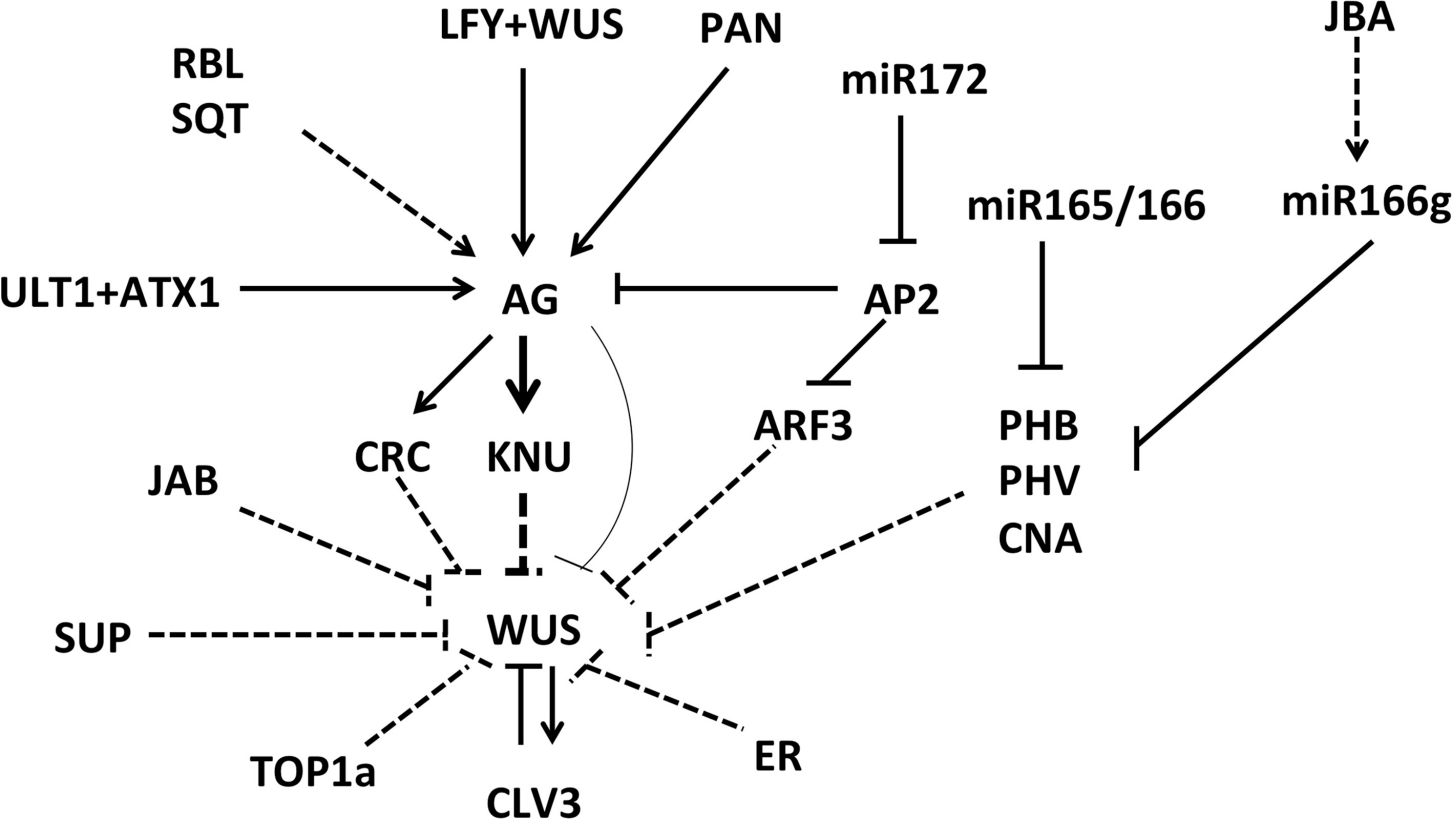
**Various factors involved in floral meristem control**. Many regulators control the expression of the stem cell identity gene *WUS* in direct and indirect ways. Solid lines indicate direct activation or repression, and dashed lines indicate a proposed type of regulation that has yet to be confirmed.

In addition to the known *CLV-WUS* signaling pathway that is responsible for the spatial maintenance of the floral stem cell niche, and in addition to the *AG-KNU-WUS* pathway for the timed termination of floral stem cells, other factors are known to be required for fine-tuning floral stem cell activities (Figure 2).

ULTRAPETALA1 (ULT1), a SAND domain containing protein (Carles and Fletcher, 2009), functions to induce *AG* in floral stem cells in an LFY-independent manner (Engelhorn et al., 2014). ULT1 may negatively regulate floral stem cell proliferation. The *ult1* mutant flowers have bigger floral meristems and prolonged *WUS* activity, resulting in five petals instead of the usual four (Fletcher, 2001). Thus, both genetic and molecular studies indicate that ULT1 negatively regulates the *WUS* expressing domain in floral buds, potentially through the *AG-WUS* regulatory pathway (Carles et al., 2004). ULT1 is reported be a trithorax group (trxG) protein that can physically interact with another trxG protein, ATX1, a H3K4me3 methyltransferase (Alvarez-Venegas et al., 2003; Carles and Fletcher, 2009). By binding directly to *AG* regulatory sequences, ULT1 may recruit ATX1 to actively modulate the methylation status of nucleosomes at the *AG* locus.

Two other factors, REBELOTE (RBL) and SQUINT (SQN), can redundantly regulate floral stem cells in addition to ULT1 (Prunet et al., 2008). Reiterative reproductive floral organs are observed in flowers of the double mutants *rbl sqn, rbl ult1*, and *sqn ult1*. In the double mutant flowers, *WUS* activity is prolonged. Presumably, *RBL* and *SQN* both regulate the floral meristem by reinforcing *AG* expression. As a cyclophilin protein, SQN was recently found to bind the protein chaperone Hsp90 and promote microRNA activity via AGO1 (Earley and Poethig, 2011). The *sqn* single mutant displays increased carpel number relative to wild-type, and it has abnormal phyllotaxy of the flowers. This phenotype increased expression of *SPL* family transcription factors, which are targeted by the microRNA miR156 (Smith et al., 2009). *PERIANTHIA* (*PAN*), a bZIP transcription factor, also affects floral stem cell activity through direct activation of *AG* (Running and Meyerowitz, 1996; Chuang et al., 1999; Das et al., 2009; Maier et al., 2009). In *pan* mutant flowers, *AG* mRNA levels are reduced in short-day conditions, resulting in flowers with an increased number of floral organs. In addition, increased floral meristem indeterminacy is observed in *lfy pan* and *seuss* (*seu*) *pan* double mutant flowers. Ectopic floral organs continue to grow inside the fourth whorl floral organs of *lfy pan* and *seu pan* plants, suggesting a potential effect of the floral identity gene *LFY* and the adaptor-like transcriptional repressor *SEU* in floral meristem regulation (Das et al., 2009; Wynn et al., 2014).

*SUPERMAN* (*SUP*), which encodes a C2H2 zinc finger protein with a C-terminal EAR-like repression motif, is thought to function as a transcriptional repressor during flower development (Hiratsu et al., 2002). Loss-of-function mutants of *SUP* produce supernumerary stamens at the expense of carpels, indicating that *SUP* has a role in maintaining the boundary between the 3rd and 4th whorl floral organs (Sakai et al., 1995). Compared to the *ag-1* mutant flowers, flowers of the double mutant *ag-1 sup* produce greatly enlarged floral meristems, generating reiterating whorls of petals, indicating the role of *SUP* in floral stem cell regulation in parallel with *AG* (Bowman et al., 1992).

*CRABS CLAW* (*CRC*), which is a direct downstream target of AG, is reported to be involved in floral meristem control. Null mutants for *crc-1* do not show floral meristem defects; instead, the apical part of the mutant carpel is unfused. However, in combination with certain other mutants, supernumerary whorls of floral organs are observed; this occurs in *crc-1 spatula-2, crc-1 ag-1/+, crc-1 rbl-1, crc-1 sqn-4, crc-1 ult1-4, crc-1 pan-3* and *crc-1 jaiba* double mutant flowers (Prunet et al., 2008; Zuniga-Mayo et al., 2012). *CRC* encodes a YABBY family transcription factor, and its expression begins in floral stage 5-6 on the abaxial side of the carpel primordia. CRC may regulate *WUS* activity in a non-cell autonomous manner (Bowman and Smyth, 1999; Lee et al., 2005).

Various microRNAs are reported to be involved in floral meristem determinacy control. For instance, *miR172* promotes termination of floral stem cells by reducing the expression of its target, *AP2* (Chen, 2004). Over-expression of a *miR172*-resistant version of *AP2* (*35S::AP2m1/3*) leads to indeterminate stamens and petals (Chen, 2004; Zhao et al., 2007). The class III HD-ZIP genes, including *PHABULOSA* (*PHB*) and *PHAVOLUTA* (*PHV*), are targeted by *miR165/166*. Over-expression of *miR165/166* in an *ag-10* background, a weak allele of *ag*, or alleles of *PHB* and *PHV* that are resistant to *miR165/166* can lead to indeterminate growth of floral organs (Ji et al., 2011). A proper balance of *PHB/PHV* and *mir165/166* is important for floral meristem determinacy control. Consistent with this, in the triple mutant of *phb phv cna*, floral carpel number is increased (Prigge et al., 2005). Similarly, enlarged shoot meristems caused by increased *WUS* expression are observed in the *jabba1-D* mutant, a dominant allele of *JABBA* (*JBA*) that produces an increased amount of *miR166g* to regulate *PHB, PHV* and *CORONA* (*CNA*) expression (Williams et al., 2005).

The ERECTA (ER) receptor kinase-mediated regulation of *WUS* expression was recently reported to be mediated by a pathway parallel to the *WUS-CLV* pathway in both SAM and FM (Mandel et al., 2014). As a secondary signaling factor, ER works together with the nuclear protein JBA to repress *WUS*. In a *jba-1D/+ er-20* double mutant background, the SAM and floral meristem are greatly enlarged, and the spiral vegetative phyllotaxy switches to whorled patterns. In the *jba-1D/+ er-20* background, *AG* is ectopically expressed at a level that produces ectopic fused carpels from the inflorescence meristem, indicating an indirect role of ER in floral meristem identity control.

Recently, a mutation in the DNA topoisomerase gene *TOPOISOMERASE1a* (*TOP1a*) was shown to increase floral meristem indeterminacy in an *ag-10* background, as the *ag-10 top1a-2* double mutant exhibits an indeterminate floral meristem (Liu et al., 2014a). In floral stem cell regulation, TOP1a may function to reduce nucleosome density, thus facilitating PcG-mediated H3K27me3 deposition on *WUS*. Mutations in another gene *AUXIN RESPONSE FACTOR 3* (*ARF3*), have also been reported to enhance the *ag-10* indeterminate phenotype (Liu et al., 2014b). Double mutant *ag-10 arf3-29* flowers produce additional floral organs that grow inside of the unfused sepaloid carpels, suggesting that *ARF3* may reinforce floral meristem determinacy through *WUS* repression. The *ARF3* locus is directly bound by AP2, indicating that AP2's role in floral stem cell regulation is also partially mediated by ARF3.

## Conclusion

The complex regulatory network controlling floral meristem development produces elegant flowers with defined numbers and whorls of floral organs, thus ensuring that plant reproduction can occur (Figure 2). With knowledge of the spatial and temporal control of floral stem cells, as well as knowledge of the many factors responsible for fine-tuning floral stem cell activity, steady progress will be made in unraveling the mysteries of floral meristem regulation. Recently developed techniques, including ChIP-seq, RNA-seq, TALENs, CRISPR/Cas9, confocal live imaging and mathematical modeling, will help to provide further insights into the intriguing nature of flower development.

### Conflict of interest statement

The authors declare that the research was conducted in the absence of any commercial or financial relationships that could be construed as a potential conflict of interest.
